# Epidemiological characteristics and some risk factors of extrapulmonary tuberculosis in Larache, Morocco

**DOI:** 10.11604/pamj.2020.36.381.24870

**Published:** 2020-08-31

**Authors:** Adil Sbayi, Amine Arfaoui, Hasna Janah, Safaa EL Koraichi, Ali Quyou

**Affiliations:** 1 Faculty of Sciences, Ibn Tofaïl University, Kenitra, Morocco,; 2 Royal Institute of Managers Training, Sale, Morocco

**Keywords:** Extrapulmonary tuberculosis, Larache, epidemiology, risk factors

## Abstract

**Introduction:**

this work aims to bring out the epidemiological characteristics of extrapulmonary tuberculosis (EPTB) in the province of Larache (Morocco) and to investigate the effect of gender and age on its localization and treatment outcome.

**Methods:**

it consists in a retrospective study based on 2962 cases of EPTB, reported during the period 2000 to 2012.

**Results:**

the mean age was 31.74 ± 18.83 years, with a median age of 26. Males are more affected by this form of tuberculosis, with a male to female sex-ratio of 1,15. The EPTB affects particularly the young population whose age is between 15 and 34 years. The pleural and lymph node localizations are the most common with 45% and 28% respectively. The statistical analysis reveals that younger patients are preferentially affected by lymph node tuberculosis whereas oldest ones are more likely to suffer from urogenital and pericardial tuberculosis. Regarding the treatment outcome, we demonstrated that age is significantly associated with the treatment outcome and that deaths occur preferentially in the oldest patients. Finally, we found out a significant association between males and pleural ETB localization, and between females and lymph node and peritoneo-itestinal ETB localizations.

**Conclusion:**

special attention must be paid to the mentioned most vulnerable categories of EPTB patients.

## Introduction

Tuberculosis is a contagious infectious disease which represents a major public health problem worldwide, with 8.6 million new cases per year worldwide in 2012 [1]. Extrapulmonary tuberculosis (EPTB) represents 20% of tuberculosis cases worldwide [2]. In Morocco, the frequency of EPTB was estimated at 49% of all tuberculosis cases in 2014 [3]. EPTB diseases result from the spread of bacilli from regional lymph lung nodes via the lymphatic and sanguine system, to other ganglia or organs such as the kidneys, the epiphyses of long bones, vertebral bodies, meninges [4]. The multiple clinical aspects of EPTB represent a diagnostic problem for practitioners, which require histological examinations (anatomical and pathological) to make compelling arguments for the diagnosis of these forms [4]. The epidemiological profile of EPTB in the province of Larache has never been brought out and very few studies have investigated the risk factors associated with this desease in Morocco. Within this scope, the work at hand aims to bring out the epidemiological characteristics of EPTB in the province of Larache (Morocco) and to investigate the influence of gender and age on the localization and the treatment outcome of EPTB.

## Methods

The present work covers the province of Larache located in the north of Morocco and whose population came close to 494 000 inhabitants in 2012. It consists in a retrospective study based on 2962 cases of EPTB, all localizations combined, reported to the Centre for Diagnosis and Treatment of Respiratory Diseases (CDTRD) of Larache during a 13 years period from January 2000 to December 2012. These cases represent all existing cases of EPTB in Larache province during the study period. They come from the provincial hospital of Larache, Ksar El Kbir hospital, various health centers, public and private pulmonologists and general practitioners in this province. All information about tuberculosis patients are contained in individual patient record folders kept at the archives of the CDTRD. The variables we were interested in are gender, age, admission date, localization (affected organ) and treatment outcome.

The population data used for the calculation of incidence rates originated from the estimations of the High Commissioner for Planning on a provincial level. The rate of incidence was obtained by multiplying the number of cases by 100 000 and dividing by the population of the province. The specific lethality for a given category was obtained by dividing the number of deaths among this category by the number of cases in the same category. The Odds ratio measures the association between the exposed categories of two dichotomous variables. We used it in order to investigate the association between males and each EPTB localization, and between males and each treatment outcome. The odds ratio is significant when the value “1” is included in the 95% interval confidence. An odds ration greater than 1 implies an association with the exposed category, whereas an odds ratio lower than 1 means an association with the non-exposed category. We also used One Factor Analysis of Variance (Anova) to analyze the relation between age and localization, and between age and treatment outcomes. When the ANOVA test is significant, the post-hoc test is performed to divide the population into groups of equal age mean.

## Results

**Figure 1:**
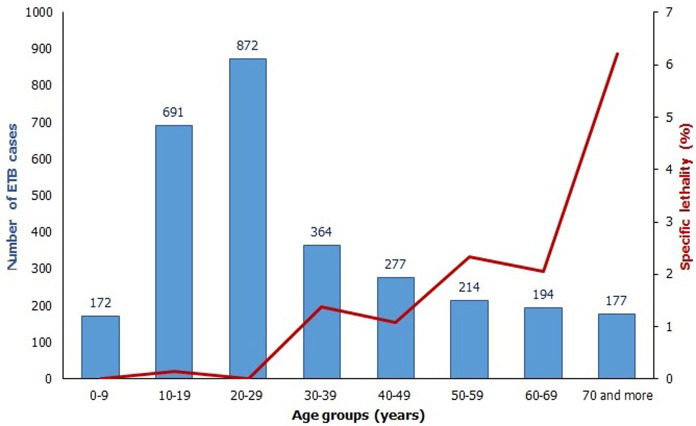
repartition of patients according to age group and specific lethality (n = 2962)

During the study period, EPTB represents 43.5% of all tuberculosis patients in Larache province, with 2962 cases including 29 deaths; that makes an overall EPTB lethality of 0.98%. The average age of patients is 31.74 ± 18.83 years, with a median age of 26 years, a minimum of six months ans a maximum of 100 years. The repartition of cases´ number and specific lethality according to age groups (Figure 1) shows that the 15-24 age group is the most affected by EPTB with 981 cases, followed by 25>-34 year-old patients with 556 cases and 35-44 year-old patients with 309 cases. Regarding the specific lethality, the maximum is registered among the oldest patients with a peak of 4.5% in 65 and more year-old patients. Male patients represent 53.4% of all cases with a sex-ratio male to female of 1.15. The mean age is 30.82 ± 18.79 years in males and 32.79 ± 18.81 years in females. This gender difference is statistically significant (t=2.84; p=0.005) which means that EPTB male patients are younger than female ones. The specific lethality is 1.2% in men and 0.7% in women

**Figure 2:**
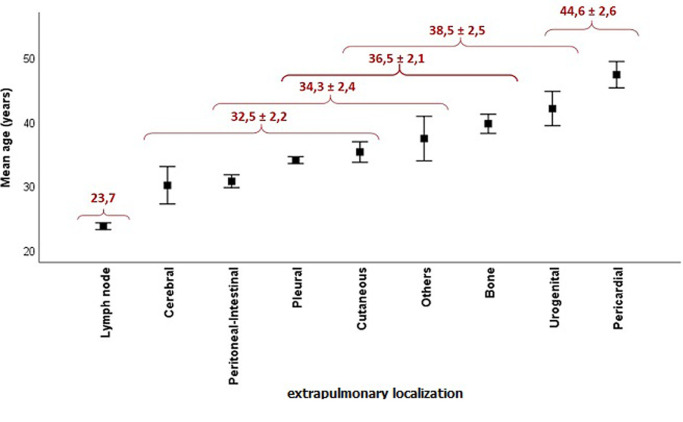
repartition of patients according to the mean age and the extrapulmonary localization (n = 2962)

**Relationship between age and EPTB localization**: the repartition of patients according to the localization of EPTB shows a predominance of pleural and lymph node tuberculosis with 45% and 28% respectively, followed by peritoneo-intestinal with 10%. Urogenital and cerebral tuberculosis are the least common with 45 and 32 cases respectively. In order to investigate the relationship between age and extrapulmonary localization, we performed an analysis of variance that showed a highly significant F ratio (F = 35.2; p<0.001), implying that the extrapulmonary localization is strongly influenced by age. The comparison of means showed the existence of six localization groups according to the age (Figure 2). The first group, with the lowest mean age (23.7 years), is made up of the lymph node localization only. The last group, with the highest mean age (44.6 ± 2.6), is composed of pericardial and urogenital localizations. The other localization makes up intermediate groups. This result means that lymph node tuberculosis affects preferentially the youngest population whereas the urogenital and pericardial tuberculosis affects the oldest one.

**Figure 3:**
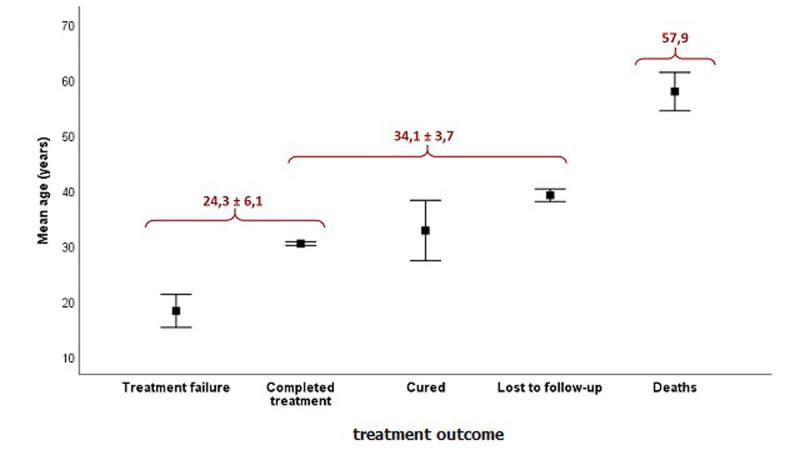
repartition of patients according to the mean age and the treatment outcome (n = 2962)

**Relationship between age and treatment outcome**: the repartition of extrapulmonary tuberculosis patients according to the treatment outcome shows that the majority of them completed treatment with 2540 cases (85.7%). Second come the lost to follow-up cases with 12.7%. Deaths represent 1% with 29 cases. The analysis of the relationship between age and treatment outcome, using the analysis of variance showed that these two variables are closely related (F = 33.9; p<0.001). The comparison of means displayed three outcome groups according to the age (Figure 3). The group with the highest mean age (57.9 years) corresponds to the dead patients. The group with the lowest mean age (24.3 ± 6.1 years) is made up of completed treatment and treatment failure outcomes. The other outcomes form an intermediate group. This result implies that the deaths in our sample preferentially affect the oldest population.

**Relationship between gender and EPTB localization**: in order to investigate the association between gender and EPTB localization, we calculated the Odds ratio (OR) for each localization by exposing males. The results in Table 1 revealed three significant OR values. Indeed, pleural tuberculosis is significantly associated with males (OR=1.68; IC95%=1.60-2.15), whereas lymph node tuberculosis (OR=0.60; IC95%=0.51-0.70) and peritoneo-itestinal tuberculosis (OR=0.54; IC95%=0.42-0.69) are significantly associated with females. That implies that men run greater risk of getting a pleural tuberculosis than women, but are less likely to suffer from lymph node or peritoneo-itestinal tuberculosis.

**Table 1:** odds ratio values between gender (males exposed) and each EPTB localization

EPTB localizations	OR (M/F)	95% Confidence Interval	
Pleural	1,86^*^	1,60	2,15
Lymph node	0,60^*^	0,51	0,70
Peritoneal-Intestinal	0,54^*^	0,42	0,69
Bone	1,21	0,88	1,65
Cutaneous	1,17	0,82	1,69
Pericardial	0,95	0,63	1,43
Urogenital	1,20	0,66	2,17
Cerebral	0,87	0,43	1,75
Others	0,73	0,37	1,43

OR: Odds ratio; ^*^: Significant value

**Relationship between gender and treatment outcomes**: the association between gender and EPTB treatment outcomes was assessed by calculating the Odds ratio (OR) for each treatment outcome, taking males as exposed. The results revealed significant OR values for completed treatment and lost to follow-up outcomes (Table 2). Indeed, completed treatment outcome is significantly associated with females (OR=0.61; IC95%=0.49-0.75), whereas lost to follow-up outcome is significantly associated with males (OR=1.59; IC95%=1.27-1.99). This result implies that women are more likely to complete their treatment than men, and are less likely to be lost to follow-up.

**Table 2:** odds ratio values between gender (males exposed) and each treatment outcome

EPTB treatment outcomes	OR (M/F)	95% Confidence Interval	
Completed treatment	0,61^*^	0,49	0,75
Lost to follow-up	1,59^*^	1,27	1,99
Deaths	1,66	0,77	3,59
Cured	2,62	0,71	9,71
Treatment failure	1,31	0,22	7,83

OR: Odds ratio; ^*^: Significant value

## Discussion

This study showed that the average age of EPTB patients was 31.74 ± 18.83 years, with a median age of 26. These values are by far lower than those reported by a study conducted in the south of Morocco, in which the average age and the median age of EPTB patients were 35 and 32 years respectively [5]. They are also lower than those found by Robert [6] Cabié [7] and Menard [8] that reported average ages of 38, 33 and 35 years respectively. Nevertheless, our finding is consistent with studies from USA, Europe and Saudi Arabia [9-11] which have found that young age was independent risk factor for EPTB. Males are more affected by this form of tuberculosis, with a male to female sex-ratio of 1.15, lower than that registered by other studies in morocco and elsewhere which is around 1.3 [5,8]. On the contrary, other studies carried out in Algeria [12] and Saudi Arabia [9] found a predominance of EPTB affection in females with a male to female sex-ratio of 0.7 and 0.9 respectively. We also showed that the patients whose age is between 15 and 34 years are the most affected by EPTB. Several studies conducted in Morocco, in Africa, in Europe and in USA have reached the same conclusion stating that the age of the majority of patients is between 20 and 40 years [5-8,10,11].

Another important result is that the pleural EPTB is the most common with 45%, followed by lymph node tuberculosis with 28%, which is consistent with other studies in morocco [5] and elsewhere [8,13,14] , but differs from other studies that place the lymph node at the forefront of EPTB localizations [9,10,15,16]. However, In Hong Kong, the genitourinary system and the skin were the most common sites [17], whereas in the USA, bones and/or joints were the most common ones [18]. These disparities could be explained by ethnic differences and also by the level of endemicity of tuberculosis [8]. Furthermore, the results showed that younger patients are preferentially affected by lymph node tuberculosis whereas the oldest ones are more likely to suffer from urogenital and pericardial tuberculosis. This is in accordance with other works for which lymph node and cerebral tuberculosis affect the youngest population whereas urogenital and bone tuberculosis affect the oldest one [19-23]. Regarding the treatment outcome, the present study displayed a predominance of patients who completed their treatment followed by those lost to follow-up. This repartition does not differ from those reported by several other studies [7,13,19,24].

The investigation of relationship between the age and the treatment outcome showed that the deaths preferentially occur in older people and that the specific lethality increases proportionally with age. In fact, it has been demonstrated that EPTB was associated with a poor immunosuppressive factor such as HIV, alcoholism and chronic disease, such as diabetes and kidney failure, which are, in most cases, expressed in old age. This may be the main cause of EPTB cases and deaths among the elderly patients, both in developing and industrialized countries [25,26]. Finally, this work allowed us to find out a significant association between males and pleural ETB localization, and between females and lymph node and peritoneo-itestinal ETB localizations. In accordance with this finding, Ghiar *et al*. [12], Noertjojo *et al*. [17] and Chan-Yeung *et al*. [27], all demonstrated a higher risk of pleural localization in males and a higher risk of lymph node localization in females. Moreover, the results showed that women are much more disciplined for treatment than men, which implies that greater effort must be made for raising men awareness in Larache of the importance of the treatment follow-up in recovery.

## Conclusion

It should be pointed out that extrapulmonary tuberculosis in Larache province remains a serious problem of public health because of its incidence and mortality rates, especially among the elderly population. Thus, more efforts must be made by health authorities in order to achieve better rates of treatment success.

### What is known about this topic

- The overall epidemiological profile of extrapulmonary TB is known for some other Moroccan regions and elsewhere;
- Several studies find out that age and gender represent risk factors for this disease.

### What this study adds

- This is the first research on tuberculosis epidemiology that was conducted on the province of Larache in Morocco, and was based on official data;
- The results showed a different age profile of TB patients compared to other Moroccan provinces.
